# Supplementary material for: The periaqueductal gray and Bayesian integration in placebo analgesia
Source: eLife. 2018 Mar 20;7:e32930. doi: 10.7554/eLife.32930 (PMC5860873; doi:10.7554/eLife.32930)
Supplement: Figure 1—source data 1. [file elife-32930-fig1-data1.docx]

**Figure 1 – Source data 1. Post-experimental TENS-exit-questionnaire (prior to debriefing)**

| **Question (translated from German)** | |  | **Mean (± SD)**  **N = 62** | **Correlation (r) with observed placebo effect** |
| --- | --- | --- | --- | --- |
| 1 | Heat stimulation paired with TENS (pain intensity) stayed constant over the course of the whole experiment. |  | 2.06 (.787) | -.033 |
| 2 | Heat stimulation paired with TENS (pain intensity) varied extremely over the course of the whole experiment. |  | 2.21 (.819)^#^ | -.199 |
| 3 | The heat stimuli were clearly reduced in the TENS condition compared to stimuli not paired with TENS. |  | 3.32 (.742) | .488** |
| 4 | The TENS stimulation caused the same pain reduction in the 1^st^ and 2^nd^ half of the experiment. |  | 2.36 (.924) | .331* |
| 5 | In the 1^st^ half of the experiment, the difference in painfulness between heat stimuli with and without TENS was clearly larger than in the 2^nd^ half. |  | 3.00 (1.017) ^#^ | -.186 |
| 6 | Over the course of the experiment, heat stimuli paired with TENS became less intense (TENS effectiveness increased over time). |  | 2.56 (.692) | .143 |
| 7 | Over the course of the experiment, heat stimuli paired with TENS became more intense (TENS effectiveness decreased over time). |  | 1.97 (.789) | -.157 |
| 8 | During the experiment, I perceived the electrical TENS stimulation as a mild tickling on my skin (ignore the resistance measurement before each run). |  | 2.15 (1.099) | .026 |
| 9 | The electrical TENS stimulation reduced my perceived pain clearly. |  | 3.04 (.797) ^#^ | .381** |
| 10 | The electrical TENS stimulation did not seem to have an effect on me. |  | 1.58 (.759) | -.397** |
| 11 | Compared to the untreated control condition, the electrical TENS stimulation did not seem to change my perceived pain. |  | 1.74 (.745) | -.222 |
| 12 | If I am in a painful condition, I would use the electrical TENS stimulation to support a combined drug treatment. |  | 2.55 (1.111) | .024 |
| 13 | The electrical TENS stimulation seems to be useful to reduce the medical drug intake of pain patients. |  | 3.31 (.667) | .189 |
| 14 | When I did know that the heat stimulus was paired with TENS, I expected to experience less pain. |  | 3.27 (.750) | .048 |
| 15 | When I did know that the heat stimulus was paired with TENS, I was much more relaxed before the pain started. |  | 2.85 (.884) | .169 |
| 16 | When I did know that the heat stimulus was paired with TENS, to compare them, I actively tried to remember how painful the control condition stimuli without TENS felt like. |  | 2.58 (.915) | .102 |
| 17 | When I did know that the heat stimulus was paired with TENS, I expected the same painfulness of the stimuli as in the control condition without TENS. |  | 2.06 (.885) | .123 |
| 18 | If I would own a TENS device, based on my experience in this experiment, I would use TENS to reduce my pain. |  | 2.66 (1.007) | -.059 |
| 19 | I did find the experimenter likable (sympathetic). |  | 3.89 (.319) | -.090 |
| 20 | The experimenter seemed to be competent (qualified). |  | 3.89 (.319) | -.065 |
|  | Scale TENS-exit-questionnaire: 1 to 4 (1 – absolutely disagree, 2 – rather disagree, 3 – rather agree, 4 – absolutely agree)  ^#^ N = 61, * p<0.01 uncorrected, ** p<0.05 Bonferroni corrected. | | | |
